# Droplet Digital PCR for the Detection of *Pseudomonas savastanoi* pv. *savastanoi* in Asymptomatic Olive Plant Material

**DOI:** 10.3390/plants14121831

**Published:** 2025-06-14

**Authors:** Giuseppe Tatulli, Nicoletta Pucci, Elena Santilli, Valeria Scala, Stefania Loreti

**Affiliations:** 1 Council for Agricultural Research and Economics, Research Centre for Plant Protection and Certification of Rome, 00156 Rome, Italy; nicoletta.pucci@crea.gov.it (N.P.); valeria.scala@crea.gov.it (V.S.); 2 Council for Agricultural Research and Economics, Research Centre for Olive, Fruit and Citrus Crops (CREA-OFA), Rende, 87036 Cosenza, Italy; elena.santilli@crea.gov.it

**Keywords:** quantitative PCR, olive knot disease, *Olea europaea*, diagnostic tools

## Abstract

Olive knot disease, caused by *Pseudomonas savastanoi* pv. *savastanoi*, severely impacts olive tree yield and oil quality. Early and accurate detection of the bacterium’s presence, particularly in asymptomatic plants, is crucial for effective disease management. This study aimed to develop an improved protocol for processing plant samples and adapting quantitative PCR to droplet digital PCR (ddPCR). For this purpose, four plant preparations—EW (external washing), PELLET (bacterial concentration), and enrichment in liquid media for 24 or 48 h (24hE, 48hE)—were tested using spiked samples. The ddPCR was set up and compared with qPCR to evaluate analytical sensitivity and specificity. Additionally, field samples from symptomatic and asymptomatic olive orchards were tested to evaluate the performance of the selected methods in naturally infected plants. ddPCR showed higher sensitivity than qPCR, particularly with the PELLET and 24hE preparations. The PELLET from the spiked sample preparation achieved a limit of detection of 10 CFU/mL for both molecular tests. The ddPCR, combined with the PELLET preparation, offers a highly sensitive and reliable tool for detecting *P. savastanoi* pv. *savastanoi* in asymptomatic olive material. This protocol shows great potential for improving early bacterial detection and disease prevention, thus aiding control strategies in nurseries and olive orchards, and supporting the production of certified plant propagation material.

## 1. Introduction

*Pseudomonas savastanoi* pv. *savastanoi* is the causal agent of olive knot disease (OKD), characterized by the formation of tumorous growths, or knots, primarily on the branches and twigs of olive trees (*Olea europaea*) [1,2], and occasionally on leaves and fruits [2,3,4,5]. OKD is a chronic disease, as symptoms persist and recur for many years in infected plants.

*P. savastanoi* pv. *savastanoi* enters the host plant through wounds resulting from various causes such as harvesting, pruning, hail, frost, and leaf scars. Knot development is driven by bacterial phytohormones, such as 3-indoleacetic acid and cytokinins, which promote uncontrolled cell growth around the infection site [6,7,8]. The knots contribute to the decline and death of branches, resulting in significant yield losses. Tree vigour, growth and the size and quality of the fruits can be moderately or severely reduced [1,9]. Crop losses caused by olive knot are not clearly assessed, and greatly depend on the geographical location and olive cultivar [4]. *P. savastanoi* pv. *savastanoi* can persist as an epiphyte on the surface of aerial plant parts and within young knots [1,10]. This persistence represents a critical inoculum source for new infections, allowing for rapid disease transmission across the entire orchard [1]. The bacterium can spread via rain, wind, insects, and human activities, such as olive grove management practices.

The dynamics of bacterial populations are influenced by seasonal factors and leaf age, with the most significant damage occurring under conditions that favor *P. savastanoi* pv. *savastanoi* epiphytic growth and its entry into olive bark, particularly during spring and autumn compared to winter and summer [10,11,12]. High rainfall and moderate temperatures (10–20 °C) are optimal for *P. savastanoi* pv. *savastanoi* epiphytic growth and penetration, resulting in significant harm to the host plant [12,13,14]. The spread of the disease through asymptomatic propagation materials is well-documented, as the epiphytic and endophytic lifestyle of *P. savastanoi* pv. *savastanoi* makes plants for planting a key pathway for its long-distance dissemination [15,16,17]. Consequently, the pathogen is usually introduced into new areas through asymptomatic infected plant material.

Controlling OKD is challenging, and current strategies focus on preventive measures such as reducing both endophytic and epiphytic bacterium populations through sanitary and cultural practices [1,3,4,18]. These strategies include pruning symptomatic branches, disinfecting the resulting wound and applying foliar treatments with copper-based compounds. Preventive measures also include the use of certified pathogen-tested plants and rootstocks for establishing new olive groves [19]. It is worth noting that the European phytosanitary certification program for the production of certified olive trees includes verification of the absence of *P. savastanoi* pv. *savastanoi* [20]. Thus, reliable diagnostic tests for detecting *P. savastanoi* pv. *savastanoi* in asymptomatic plant material are essential. Conventional *P. savastanoi* pv. *savastanoi* diagnosis relies on visual inspection of typical symptoms (e.g., knots) and direct bacterial isolation followed by pathogenicity and biochemical or serological tests [21,22,23,24]. However, these methods are time-consuming and lack sensitivity and specificity, especially for asymptomatic plants, where bacterial isolation is often unreliable [25,26,27,28,29]. To overcome these limitations, several assays for *P. savastanoi* pv. *savastanoi* detection have been developed, including PCR [26], nested PCR [29], enriched PCR [26], qPCR [27], and High-Resolution Melting Analysis (HRMA) [30]. These tools have demonstrated sensitivity and specificity, as well as the ability to discriminate among different pathovars (i.e., *savastanoi*, *nerii*, *fraxini*) or to quantify the pathogen. Despite this, a low bacterial load and/or the presence of inhibitors in plant matrices may lead to false negatives even with molecular methods. Under these conditions, two steps could be improved: (i) plant material processing to increase the detectable bacterial load and (ii) molecular tests less affected by inhibitors.

Bertolini et al. (2003) [28] compared two methods for processing plant material: external washing versus washing followed by an enrichment step. Their findings showed that the enrichment step enhanced the detection efficiency of *P. savastanoi* pv. *savastanoi*, both as an epiphyte and an endophyte. In this study, the initial steps for processing plant material were optimized to improve bacterial DNA detection by increasing sensitivity and minimizing PCR inhibition. To achieve this, the protocol described by Bertolini et al. 2003 [28] was partially modified by adding a concentration step after washing and by reducing the enrichment time from 72 h to 24 or 48 h. DNA was then extracted using four different approaches: (i) external washing step (EW); (ii) pellet of concentrated washing (PELLET); (iii) 24 h enrichment (24hE); and (iv) 48 h enrichment (48hE). These preparations were tested using both qPCR [27] and droplet digital PCR (ddPCR). The ddPCR has shown promise for efficiently detecting pathogens such as *Ralstonia solanacearum* in potatoes [31] and *Xylella fastidiosa* spp. in several plant hosts [32], showing sensitivity comparable to qPCR. Other studies showed increased detection sensitivity of ddPCR compared to qPCR for *Xanthomonas citri* subsp. *citri* [33,34], pepper mild mottle virus in plants, soil, and water [35], and *Phytophthora nicotianae* in environmental samples [36]. Recently, a ddPCR protocol for *X. fastidiosa* detection was implemented and showed greater analytical sensitivity than qPCR for *O. europea, C. sinensis,* and *N. oleander* [37]. The ddPCR allows the detection and quantification of pathogens, such as *Agrobacterium vitis* in grapevines, for which previous methods lacked sensitivity [38]. Unlike qPCR, ddPCR provides absolute target quantification without the need for a standard curve, facilitating data comparison and experimental design when standards are unavailable [38]. Additionally, ddPCR is less affected by PCR inhibitors than qPCR [38,39]. Given the challenges of detecting *P. savastanoi* pv. *savastanoi* in asymptomatic samples, using ddPCR as a highly sensitive and specific diagnostic method, this study aimed to develop a protocol for preparing samples and adapting the qPCR method by Tegli et al. 2010 [27] to ddPCR. To achieve this, ddPCR was first developed using spiked samples and then tested on naturally infected asymptomatic samples collected from olive trees in different Italian regions. The developed procedure showed high sensitivity, representing a valid diagnostic tool for testing the phytosanitary status of olive plant material.

## 2. Results

### 2.1. Optimization of the ddPCR Assay

**Figure 1:**
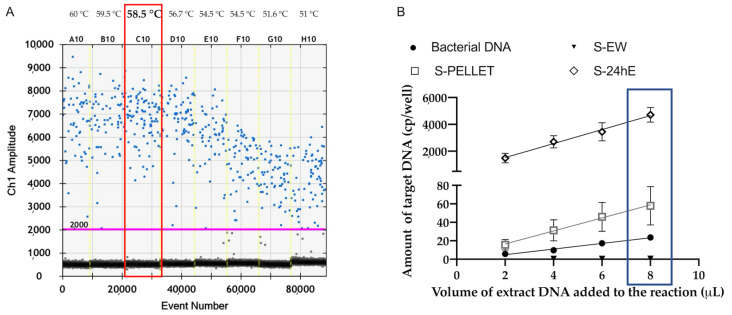
ddPCR optimization: (**A**) The solid pink line represents the threshold, with blue droplets above considered positive, indicating PCR amplification, and grey droplets below considered negative, indicating no amplification. The red box indicates the optimal annealing temperature. Eight ddPCR reactions, each with the same amount of target DNA, are separated by vertical dotted yellow lines. The reactions were performed across an annealing temperature gradient of 51 °C, 51.6 °C, 54.5 °C, 55.4 °C, 56.7 °C, 58.5 °C, 59.5 °C, and 60 °C. (**B**) ddPCR linear correlation graph illustrating the relationship between the detected DNA target concentration (*y*-axis) and the reaction DNA volume (*x*-axis).

Among the tested annealing temperatures, 58 °C was selected as the optimal temperature due to the highest fluorescence amplitude, minimal rain, and the greatest separation between negative and positive droplet clouds (Figure 1A). Following the identification of the optimal annealing temperature for ddPCR, the analytical sensitivity and inhibitory potential of the tested matrices were evaluated using DNA extracted from pure *P. savastanoi* pv. *savastanoi* colonies and spiked samples (S-EW, S-PELLET, S-24hE, and S-48hE) at a known concentration (10^3^ CFU/mL).

Analytical sensitivity improved linearly with increasing DNA volumes in the reaction mix for both S-PELLET and S-24hE samples (Figure 1B, Table 1). In contrast, DNA extracted from the S-EW fraction did not show a linear increase in target detection, likely due to the low target concentration, while the S-48hE reaction was saturated, preventing accurate ddPCR analyses. Under these conditions, the correlation coefficient was calculated to assess the relationship between the amount of target DNA and the volume of DNA used in the ddPCR reaction. As shown in Table 1, S-PELLET and S-24hE exhibit a high degree of correlation between the amount of target DNA and the increased DNA volume per reaction, making them the most suitable extraction methods for analysing asymptomatic samples. Additionally, 8 μL of DNA per ddPCR reaction represents the optimal volume for maximizing the method’s sensitivity (Figure 1B). Moreover, the ddPCR reaction was not affected by plant matrix inhibitors.

### 2.2. ddPCR Analytical Specificity

The evaluation of analytical specificity performed for both qPCR and ddPCR yielded the same results (Table 2). All non-target strains tested showed negative results with both tests, while only the *P. savastanoi* pv. *savastanoi* target strains from olive and oleander tested positive.

### 2.3. ddPCR Analytical Sensitivity

The limit of detection (LoD) of the qPCR and ddPCR in all the spiked (S) samples (S-EW, PELLET, 24hE, and 48hE) was assessed (Table 3). Both methods exhibited the lowest analytical sensitivity when testing S-EW, with a LoD of 10^4^ CFU/mL. Conversely, both methods showed high sensitivity for S-PELLET, achieving a LoD of 10 CFU/mL. For S-24hE, ddPCR displayed a 10-fold higher LoD than qPCR (10 CFU/mL vs. 10^2^ CFU/mL). However, ddPCR results for S-48hE were saturated, making this preparative method unsuitable, while qPCR maintained a high sensitivity with a LoD of 10 CFU/mL. No false positives were observed.

### 2.4. Field Samples

All four plant material preparations from field (F) samples (F-EW, F-PELLET, F-24hE, F-48hE) were preliminarily assessed on 20 out of 100 samples. The results showed that F-EW was less reliable, while F-48hE showed a lower percentage of positive samples (20%) compared to F-24hE (50%).

Based on these preliminary results and those from spiked samples, the PELLET and the 24hE preparations were selected and evaluated on asymptomatic samples collected in fields infected by *P. savastanoi* pv. *savastanoi*. Table 4 presents the cumulative results for all tested samples, showing a higher sensitivity of ddPCR compared to qPCR, both for F-PELLET (34% vs. 19%) and for F-24hE samples (22% vs. 3%). The Chi-square test used to compare the distribution of positive samples between the two detection methods revealed a significant difference (χ^2^ = 4.771, df = 1, *p* = 0.0289*), suggesting that ddPCR has higher sensitivity than qPCR, particularly in the F-24hE preparation.

**Figure 2:**
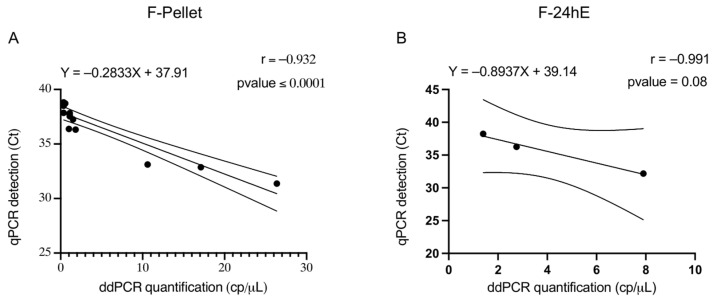
Correlation analysis of qPCR and ddPCR results for F-PELLET (**A**) and F-24hE (**B**) preparations. The solid line represents the linear regression line. The dashed lines indicate the 95% confidence interval of the regression.

Pearson’s correlation analysis was performed on F-PELLET and F-24hE samples that yielded positive results in both qPCR and ddPCR (Figure 2A,B). In both cases, a high degree of correlation was observed (F-PELLET r = −0.932; F-24hE r = −0.991). However, while the correlation value for the PELLET samples is statistically significant (*p*-value < 0.0001; n = 14), the correlation for the 24hE samples is not statistically significant (*p*-value < 0.08) due to the low number of positive results obtained in qPCR (n = 3) (Table 4).

The results of qPCR and ddPCR were analysed based on the sampling period. The percentage of positive samples detected during the two sampling periods is reported in Table 5. The sampling in April-May, combined with the use of ddPCR, seems more efficient in detecting *P. savastanoi* pv. *savastanoi*, with respect to October-November, with 40% of F-PELLET samples and 31% of F-24hE samples tested positive. Conversely, the use of qPCR as a detection method does not highlight differences between the two sampling periods. These differences were analysed using the Chi-square test, which, however, did not show a statistically significant difference between the methods across the two sampling periods (April/May: χ^2^ = 1.891, df = 1, *p* = 0.1691; October/November: χ^2^ = 1.891, df = 1, *p* = 0.1691). (April/May χ^2^ = 0.9122, df = 1, *p* = 0.3395; October/November χ^2^ = 2.632, df = 1, *p* = 0.1048).

## 3. Discussion

Olive knot disease is known for causing severe damage to olive trees, significantly affecting both the yield and quality of olive oil, with substantial economic consequences for the Mediterranean olive oil industry [2].

The pathogen’s ability to persist, even in a low concentration, in asymptomatic olive material represents a critical challenge for the management of OKD and for the detection of the causal agent *P. savastanoi* pv. *savastanoi*. This bacterium thrives in the Mediterranean climate, where conditions such as high humidity and moderate temperatures promote its growth [12,13,14].

Conventional control strategies for OKD primarily focus on reducing bacterial populations through physical and chemical means. Pruning infected branches helps remove visible sources of infection [3], while traditional copper-based treatments or innovative approaches, such as copper-nanoparticles, thyme essential oil nanoparticles [40,41], or essential oils [42] aim to reduce the bacterial load. In addition to implementing control measures, early and accurate detection of *P. savastanoi* pv. *savastanoi* is essential to prevent the spread and introduction in new areas of *P. savastanoi* pv. *savastanoi*. Therefore, the application of efficient and reliable diagnostic methods becomes crucial. The current diagnostic tools, including visual inspection, isolation and qPCR, have limitations. Visual inspection and bacterial isolation can be unreliable, especially in asymptomatic plants where disease symptoms are not yet evident and bacterial load is generally low. Although qPCR is a highly sensitive molecular test, it can be not efficient to detect low bacterial loads in asymptomatic samples and may be affected by inhibitors present in plant matrices [43,44,45]. This highlights the need for more reliable diagnostic tools that can detect the pathogen before it leads to noticeable symptoms and, consequently, damage.

In this context ddPCR shows increased sensitivity and specificity by partitioning the sample into thousands of droplets, allowing for precise quantification of target DNA without relying on a standard curve [38,45]. This makes ddPCR particularly suited for detecting pathogens at low concentrations, which is essential for identifying latent infections in asymptomatic plants [36,46].

This study focused on the optimization of the procedure for the detection of *P. savastanoi* pv. *savastanoi* from an asymptomatic olive plant, by improving sample preparation and by developing a sensitive ddPCR to enhance diagnostic reliability.

Among the four evaluated preparations on spiked samples, the S-PELLET and S-24hE proved to be the most effective in terms of analytical sensitivity. Furthermore, the S-PELLET, which involves concentrating the bacterial load of the washing solution, showed higher sensitivity achieving a high limit of detection of 10 CFU/mL for both qPCR PCR and ddPCR. Conversely, the S-EW exhibited lower sensitivity, likely due to the low bacterial load in the washing solution fraction. The S-48hE preparation, while effective for *P. savastanoi* pv. *savastanoi* detection by qPCR, showed ddPCR saturation, even at the lower concentration of 10 CFU/mL, making it less suitable for ddPCR.

Regarding the two selected sample preparation methods (F-PELLET and F-24hE) used for the analysis of field samples, ddPCR demonstrated higher efficiency than qPCR in detecting *P. savastanoi* pv. *savastanoi* in 100 asymptomatic samples collected from both symptomatic and asymptomatic olive trees. These results highlight the improved analytical sensitivity of ddPCR, especially under challenging conditions where bacterial concentrations may be low, unevenly distributed, or where cells may be in a viable but non-culturable (VBNC) state—common scenarios in asymptomatic plant tissues.

Unlike spiked samples, field samples present greater variability. Such variability can affect both the presence and composition of PCR inhibitors. In this context, ddPCR offers a clear advantage over qPCR, as its droplet-based partitioning reduces the impact of inhibitors and enhances the detection of low bacterial DNA concentrations, thereby increasing overall accuracy. The higher efficiency of ddPCR in detecting the F-PELLET samples can be also attributed to its ability to detect DNA from both viable and non-viable bacterial cells, with respect to F-24hE preparation that enriched only viable cells. The concentration step increases the likelihood of capturing bacterial cells, even when the bacterial load is low. This makes it particularly useful for detecting *P. savastanoi* pv. *savastanoi* in asymptomatic samples, where bacteria may be present in low numbers or in a latent state. Conversely, the F-24hE, which requires viable bacterial cells to grow in an enrichment medium, was less effective. The F-24hE may be less effective than F-PELLET also due to the presence of *P. savastanoi* pv. *savastanoi* in a VBNC state. *P. savastanoi* pv. *savastanoi* can switch from a viable to a VBNC state, i.e., in plants exposed to heavy metal copper ions [18]. However, the F-24hE procedure still provided valuable insights when combined with ddPCR.

Pearson’s correlation coefficient for PELLET samples was statistically significant, indicating a strong correlation between the results obtained with the two molecular methods. This high correlation supports the reliability of ddPCR as an alternative to qPCR, with the added benefit of increased efficiency and sensitivity.

Given the seasonality of *P. savastanoi* pv. *savastanoi*’s activity and epiphytic growth we investigated if monitoring activity may be affected by the season of sampling. Our results highlighted a higher efficiency of ddPCR compared to qPCR, particularly during April/May for both sample preparation methods (F-PELLET: 40% vs. 12%; F-24hE: 31% vs. 4%). Nevertheless, these differences were not statistically significant. During the October/November sampling period, ddPCR also showed higher performance than qPCR, although the differences were less pronounced compared to the April/May period (F-PELLET: 26% vs. 20%; F-24hE: 13% vs. 2%). Similarly, these differences were not statistically significant.

In conclusion, the findings of the spiked and the field samples confirm that ddPCR is a highly sensitive and reliable tool for detecting *P. savastanoi* pv. *savastanoi* in olive trees, particularly in asymptomatic plant material. The PELLET preparation, in combination with ddPCR, offers the most effective procedure. On the other hand, the 24hE can be useful when the detection of only viable cells is advisable. Together, these results highlight the potential of ddPCR to improve early detection and management of OKD, ultimately contributing to the prevention and limiting the spread of this harmful pathogen through asymptomatic plant propagation material.

## 4. Materials and Methods

### 4.1. Bacterial Strains

The *P. savastanoi* pv. *savastanoi* strain (CREA-DC collection number 1918, CREA-DC, Rome, Italy), isolated in 2018 from an olive plant affected by olive knots in the Lazio region, was routinely cultured at 28 °C on nutrient agar (Oxoid, CM0003, Altrincham, UK) added of 0.25% glucose (NAG). The colony-forming units (CFUs) were quantified by plating 100 μL of bacterial suspensions on NAG medium and incubating at 28 °C for 4 days. The optical density of 0.1 at 600 nm corresponded to 10^7^ CFU/mL, as reported by Martinez et al. in 2010 [47].

### 4.2. Spiked Sample Preparation

Samples were processed following Bertolini et al. [28] with modifications. Asymptomatic twigs were collected from olive plants previously tested by qPCR of Tegli et al. [27] to verify the absence of *P. savastanoi* pv. *savastanoi*. Twenty grams of young, leafless twigs were cut into ~5 cm fragments and placed in 100 mL of PBS1X with 0.05% Tween-20 (Sigma Aldrich, St. Louis, MO, USA) containing *P. savastanoi* pv. *savastanoi* bacterial suspensions at final concentrations of 10^4^, 10^3^, 10^2^, and 10 CFU/mL. The washing solution from each spiked sample was divided into four preparations for DNA extraction: (i) external wash (S-EW); (ii) 96 mL of external wash was centrifugated at 10,000× *g* for 20 min at 4 °C, with the resulting pellet resuspended in 5 mL of PBS1X (S-PELLET); (iii) 500 μL of the external wash enriched in 4.5 mL of PVF-1 semi-selective medium for 24 h (S-24hE); (iv) 500 μL of the external wash enriched in 4.5 mL of PVF-1 for 48 h (S-48hE). PVF-1 medium used for enrichment analyses was prepared following Surico and Lavermicocca [22]. Three independent biological replicates were prepared for each preparation and each bacterial concentration.

### 4.3. Analytical Sensitivity

Analytical sensitivity of ddPCR was evaluated in comparison with qPCR Tegli et al. [27] following the EPPO Standard 7/98 (5) [48]. This was achieved by determining the lowest cell density that yielded a positive result, using three independent biological replicates of spiked samples.

### 4.4. Field Samples Collection

**Figure 3:**
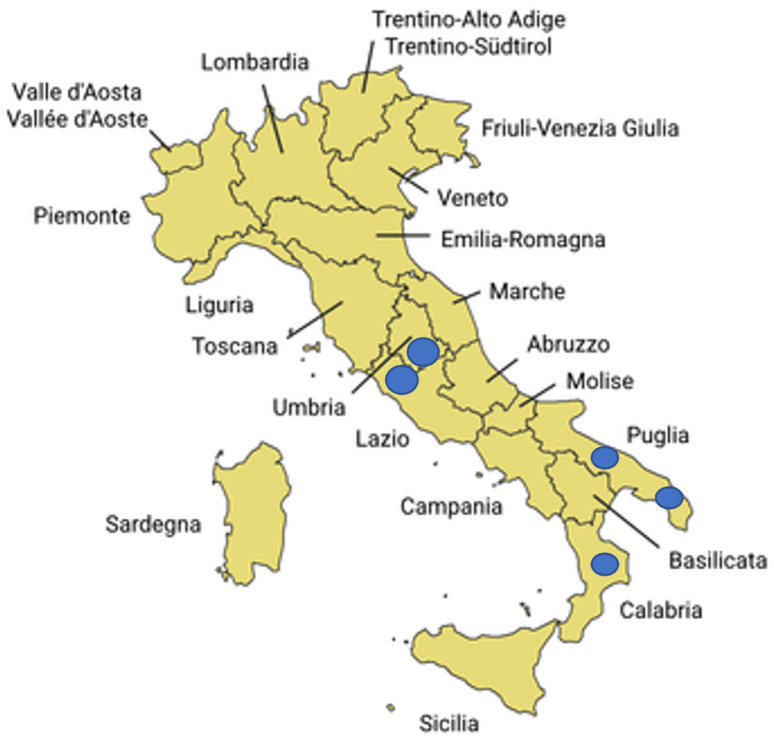
Map of Italian regions included in the sampling; the dots indicate the sampling regions.

One hundred asymptomatic samples were collected from several Italian regions known for their extensive olive-growing tradition (Puglia, Calabria, Lazio, Umbria) (Table 6 and Figure 3) during spring and autumn (April, May, October, and November 2022). In particular, 24 samples were collected in Central Italy (Lazio and Umbria) and 76 in South of Italy (Apulia and Calabria); 52 samples were collected in spring (April–May) and 48 in autumn (October–November).

The asymptomatic samples were collected from knotless branches of plants showing symptoms of *P. savastanoi* pv. *savastanoi* infection and from plants adjacent to symptomatic ones. Each sample consisted in twenty grams of young, leafless twigs, cut into ~5 cm fragments. Twenty out of 100 samples were preliminarily processed as described for spiked samples with the four plant material preparations (F-EW, F-PELLET, F-24hE, F-48hE). Based on the results obtained, the remaining 80 samples were tested using the selected F-PELLET and F-24hE preparations.

### 4.5. DNA Extraction

DNA extraction from spiked and field-collected samples was performed using two different extraction kits, depending on the sample type. A total of 500 μL of the bacterial suspension (positive control), EW, 24hE, and 48E samples were extracted using the Gentra Puregene Yeast/Bact. Kit B (Qiagen, PL Venlo, The Netherlands); 500 μL of resuspended PELLET were extracted using the DNeasy Plant Mini kit (Qiagen, PL Venlo, The Netherlands). The DNA was stored at −15 °C.

### 4.6. qPCR and Droplet Digital PCR

The qPCR was carried out using the thermal cycler CFX96 real-time System C1000 Touch (Bio-Rad Laboratories Inc., Hercules, CA, USA) following Tegli et al. 2010 [27]. Primers and probe specific for *P. savastanoi* pv. *savastanoi* were used to develop the ddPCR reaction using the QX200^TM^ Droplet Digital^TM^ PCR System (Bio-Rad, Hercules, CA, USA) in accordance with the manufacturer’s instructions. The reaction included 2xddPCRTM Supermix for Probes no dUTP (Bio-Rad Laboratories Inc., Hercules, CA, USA). Droplets were generated using a Droplet Generator (DG) with an 8-channel DG8 cartridge and cartridge holder with 70 μL of DG oil/well, 20 μL of PCR reaction mix and a DG8 gasket. Subsequently, the droplets (40 μL) were transferred to corresponding wells of a 96-well PCR plate (Bio-Rad Laboratories Inc., Hercules, CA, USA) and heat-sealed with pierceable foil using a PX1TM PCR plate sealer (Bio-Rad Laboratories Inc., Hercules, CA, USA) before amplification on the thermal cycler CFX96 Real-time System C1000 Touch (Bio-Rad Laboratories Inc., Hercules, CA, USA). The thermal cycling conditions were optimized using an annealing gradient ranging from 51 °C to 60 °C. The ddPCR assay was performed on DNA extracted from each spiked preparation (S-EW, S-PELLET, S-24hE) as well as from bacterial DNA at a concentration of 10^3^ CFU/mL. For each preparation, different DNA volumes (2 μL, 4 μL, 6 μL, and 8 μL) were tested in the reaction mix. The optimal reaction conditions were identified testing three parameters: (i) achieving the highest fluorescence amplitude; (ii) ensuring clear separation between positive and negative droplets; (iii) minimizing rain (i.e., droplets ranging between the positive and negative ones). The optimized thermal cycling conditions consisted of an initial denaturation step at 95 °C for 10 min, followed by 40 cycles of a two-step thermal profile: 30 s at 94 °C for denaturation and 1 min at 58 °C for annealing/extension. This was followed by a final hold at 98 °C for 10 min for droplet stabilization and cooling to 4 °C. A temperature ramp of 2.5 °C/s was applied during all PCR steps, and the lid temperature was maintained at 105 °C, as recommended by Bio-Rad. At the end of the amplification, the 96-well plate was transferred to the QX200™ Droplet Reader (Bio-Rad Laboratories Inc., Hercules, CA, USA. The ddPCR reactions generating less than 10,000 droplets were excluded from the analysis. A sample was considered positive when at least two positive droplets were detected. All qPCR and ddPCR runs included a positive amplification control (PAC; DNA from 10^6^ CFU/mL bacterial suspension), and a negative amplification control (NAC; PCR-grade water). The ddPCR data were analysed using QuantaSoft Analysis Pro software 1.0 (Bio-Rad Laboratories Inc., Hercules, CA, USA). The threshold was manually set above the negative droplet cloud, based on the results of the corresponding NAC.

The ddPCR reactions were performed on DNA extracted from samples (bacterial or spiked DNA) at concentrations up to 10^3^ CFU/mL, as higher concentrations resulted in saturation and consequent errors in Poisson statistics.

### 4.7. Analytical Specificity

The analytical specificity of the ddPCR was evaluated in comparison with qPCR following the EPPO Standard 7/98 (5) [48], on several *P. savastanoi* pv. *savastanoi* isolates, bacteria taxonomically related to *P. savastanoi* pv. *savastanoi*, olive plant epiphytes, and other plant-pathogenic bacteria (Table 7).

### 4.8. Statistical Analysis

The linear regression and Pearson’s correlation of the Cq values from qPCR, the concentration of the copy number from ddPCR, and the Chi-square test analyses were carried out using GraphPad Prism 10 Software (GraphPad Software, San Diego, CA, USA). *p*-values less than 0.05 were considered significant. Data are expressed as mean ± standard deviation (SD).

## Figures and Tables

**Table 1:** Curve information for 10^3^ spiked samples and the correlation between qPCR—ddPCR.

Samples	Curve Equation	R^2^	Correlation	*p*-Value
External wash	y = 0.03x + 0.225	0.41	0.64	0.35
PELLET	y = 7.05x + 2.42	0.9969	0.9984	0.0016 **
Enrichment 24 h	y = 597x − 348	0.9637	0.9817	0.0016 **
Bacterial DNA	y = 32.27x − 13.63	0.977	0.9884	0.0116 *

* *p*-value < 0.05; ** *p*-value < 0.02.

**Table 2:** Analytical specificity tested on several target and non-target bacterial strains (+ and − refer to test results).

Bacterial Species	Host	qPCR ^1^	ddPCR ^2^
*Pseudomonas savastanoi* pv. *savastanoi*	*Olea europaea*	+	+
*Pseudomonas savastanoi* pv. *savastanoi*	*Nerium oleander*	+	+
*Pseudomonas savastanoi* pv. *savastanoi*	*Punica granatum*	+	+
Epiphytes	*Olea europaea*	−	−
*Pantoea agglomerans*	*Cucumis melo*	−	−
*Pseudomonas marginalis* pv. *marginalis*	*Olea europaea*	−	−
*Pseudomonas syringae* pv. *syringae*	*Actinidia chinensis*	−	−
*Pseudomonas syringae* pv. *actinidiae*	*Actinidia deliciosa*	−	−
*Pseudomonas viridifava*	*Actinidia chinensis*	−	−
*Pantoea agglomerans*-like	*Olea europaea*	−	−
*Xylella fastidiosa* subsp. *pauca*	*Olea europaea*	−	−
*Xylella fastidiosa* subsp. *multiplex*	*Prunus amygdalus*	−	−
*Xanthomonas arboricola* pv. *juglandis*	*Juglans regia*	−	−
*Erwinia nigrifluens*	*Juglans regia*	−	−
*Pseudomonas syringae* pv. *glycinea*	*Glycine max*	−	−
*Xanthomonas campestris* pv. *pelargoni*	*Pelargonium*	−	−
*Erwinia billingiae*	*Pyrus comunis*	−	−
*Xanthomonas arboricola* pv. *corylina*	*Corylus avellana*	−	−
*Pseudomonas savastanoi* pv. *nerii*	*Nerium oleander*	−	−
*Pseudomonas avellanae*	*Corylus avellana*	−	−
*Xanthomonas arboricola* pv. *pruni*	*Prunus persica*	−	−
*Agrobacterium vitis*	*Vitis vinifera*	−	−

^1^ qPCR of Tegli et al. 2010 [27]; ^2^ This work.

**Table 3:** Analytical sensitivity of spiked samples obtained by qPCR and ddPCR. The LOD values for each method are shown in bold.

	S-EW		S-PELLET		S-24hE		S-48hE	
CFU/mL	qPCR ^1^	ddPCR ^2^	qPCR ^1^	ddPCR ^2^	qPCR ^1^	ddPCR ^2^	qPCR ^1^	ddPCR ^2^
1 × 10^4^	**34.9 ± 0.47 ^a^** (9) ^b^	**5.46 ± 0.44 ^c^** (3) ^d^	28.3 ± 0.53 (9)	N.P. ^e^	25.5 ± 0.27 (9)	N.P. ^e^	18.6 ± 0.84 (9)	N.P. ^e^
1 × 10^3^	38.5 ± 1.07 (7)	0.47 ± 0.44 (2)	31.9 ± 0.65 (9)	57.9 ± 20.7 (3)	29.5 ± 0.99 (9)	3342 ± 2169 (3)	20.7 ± 2.5 (9)	Saturated
1 × 10^2^	38.7 ± 0.81 (3)	0.32 ± 0.38 (2)	33.1 ± 0.72 (9)	9.25 ± 3.78 (3)	**34.9 ± 1.24** (9)	1444 ± 858 (3)	20.6 ± 2.39 (9)	Saturated
1 × 10^1^	N.A.(0)	N.A.(0)	**35.8 ± 0.41** (9)	**1.26 ± 0.80** (3)	N.A.(0)	**2232 ± 446** (3)	**20.5 ± 1.08** (9)	Saturated
Healty matrix	N.A. ^f^(0)	N.A. ^f^ (0)	N.A. ^f^ (0)	N.A. ^f^ (0)	N.A. ^f^ (0)	N.A. ^f^ (0)	N.A. ^f^ (0)	N.A. ^f^ (0)

^1^ qPCR of Tegli et al. 2010 [27]; ^2^ this work; ^a^ average Ct values ± Standard Deviation (SD); ^b^ number of positive replicates on 9 replicates analysed; ^c^ average target concentration per well ± Standard Deviation (SD); ^d^ number of positive replicates on 3 replicates analysed; ^e^ N.P., not performed; ^f^ N.A., not assigned;

**Table 4:** Positive samples obtained in qPCR and in ddPCR on a total of 100 analysed samples, for both F-PELLET and F-24hE preparations. The results are reported in percentage (%).

	qPCR ^1^	ddPCR ^2^
F-PELLET	19	34
F-24hE	3	22

^1^ qPCR of Tegli et al. 2010 [27]; ^2^ This work.

**Table 5:** Percentage of positive samples obtained by qPCR and ddPCR for F-PELLET and F-24hE samples in April–May and October–November sampling periods.

	qPCR ^1^	ddPCR ^2^
April/May		
F-PELLET	12	40
F-24hE	4	31
October/November		
F-PELLET	20	26
F-24hE	2	13

^1^ qPCR of Tegli et al. 2010 [27]; ^2^ This work.

**Table 6:** Locations and GPS coordinates of the field sampling sites.

Italian Region	Location	GPS Coordinates (Lat/Long)
Umbria	Narni	42.47861° N, 12.48934° E
Lazio	Tarquinia	42.25057° N, 11.76567° E
Lazio	Canale Monterano	42.12377° N, 12.09327° E
Puglia	Galatone	40.13748° N, 18.04628° E
Puglia	Molfetta	41.18308° N, 16.57006° E
Calabria	Rende	39.36627° N, 16.22841° E

**Table 7:** Bacterial strains used in this work.

Bacterial Species	Collection Number	Host	Year of Isolation	Countries of Isolation
*Pseudomonas savastanoi* pv. *savastanoi*	CREA-DC 1918	*Olea europaea*	2018	Italy
*Pseudomonas savastanoi* pv. *savastanoi*	NCPPB 3869	*Nerium oleander*	1985	Italy
*Pseudomonas savastanoi* pv. *savastanoi*	CREA-DC 2031	*Punica granatum*	2021	Italy
Epiphytes	CREA-DC 1936	*Olea europaea*	2019	Italy
*Pantoea agglomerans*	CFBP 6915	*Cucumis melo*	1993	Brazil
*Pseudomonas marginalis* pv. *marginalis*	CREA-DC 1229	*Olea europaea*	2001	Italy
*Pseudomonas syringae* pv. *syringae*	CREA-DC 1231	*Actinidia chinensis*	2001	Italy
*Pseudomonas syringae* pv. *actinidiae*	CREA-DC 1463	*Actinidia deliciosa*	2009	Italy
*Pseudomonas viridifava*	CREA-DC 1819	*Actinidia chinensis*	2010	Italy
*Pantoea agglomerans*-like	CREA-DC 1947	*Olea europaea*	2019	Italy
*Xylella fastidiosa* subsp. *pauca*	CFBP 8402	*Olea europaea*	2014	Italy
*Xylella fastidiosa* subsp. *multiplex*	CFBP 8730	*Prunus amygdalus*	2019	Italy
*Xanthomonas arboricola* pv. *juglandis*	CREA-DC 1020	*Juglans regia*	1992	New Zealand
*Erwinia nigrifluens*	NCPPB 564	*Juglans regia*	1958	USA
*Pseudomonas syringae* pv. *glycinea*	IPV-BO-2116	*Glycine max*	1985	Italy
*Xanthomonas campestris* pv. *pelargonii*	CREA-DC 1234	*Pelargonium*	2001	Italy
*Erwinia billingiae*	NCPPB 661	*Pyrus comunis*	1958	UK
*Xanthomonas arboricola* pv. *corylina*	NCPPB 3870	*Corylus avellana*	1991	Italy
*Pseudomonas savastanoi* pv. *nerii*	ITM 306	*Nerium oleander*	n.i.	Italy
*Pseudomonas avellanae*	CREA-DC 1110	*Corylus avellana*	1998	Italy
*Xanthomonas arboricola* pv. *pruni*	CREA-DC 1224	*Prunus persica*	2001	Italy
*Agrobacterium vitis*	CREA-DC 1822	*Vitis vinifera*	2013	Italy

n.i.,not indicated. CREA-DC, Consiglio per la Ricerca in Agricoltura, Centro di Ricerca per la Difesa e la Certificazione, Roma, Italy; NCPPB, National Collection of Plant Pathogenic Bacteria, York, UK; CFBP, Collection Française de Bactéries Phytopathogènes, INRA, Angers, France; ITM, Culture collection of Istituto Tossine e Micotossine da Parassiti vegetali, C.N.R., Bari, Italy; IPV-BO, Culture Collection of Istituto di Patologia Vegetale, Università di Bologna, Italy.

## Data Availability

Data will be available upon demand.
